# Identification of patients with cancer with a high risk to develop delirium

**DOI:** 10.1002/cam4.1106

**Published:** 2017-07-07

**Authors:** Elisabeth C. W. Neefjes, Maurice J. D. L. van der Vorst, Bertha A. T. T. Verdegaal, Aartjan T. F. Beekman, Johannes Berkhof, Henk M. W. Verheul

**Affiliations:** ^1^ Department of Medical Oncology VU University Medical Center/Cancer Center Amsterdam Amsterdam the Netherlands; ^2^ Department of Internal Medicine Rijnstate Hospital Arnhem the Netherlands; ^3^ Department of Psychiatry and EMGO Institute for Health and Care Research VU University Amsterdam the Netherlands; ^4^ Department of Epidemiology and Biostatistics VU University medical center Amsterdam the Netherlands

**Keywords:** Cancer, delirium, epidemiology, medical oncology, prevention, risk factors

## Abstract

Delirium deteriorates the quality of life in patients with cancer, but is frequently underdiagnosed and not adequately treated. In this study, we evaluated the occurrence of delirium and its risk factors in patients admitted to the hospital for treatment or palliative care in order to develop a prediction model to identify patients at high risk for delirium. In a period of 1.5 years, we evaluated the risk of developing delirium in 574 consecutively admitted patients with cancer to our academic oncology department with the Delirium Observation Screening Scale. Risk factors for delirium were extracted from the patient's chart. A delirium prediction algorithm was constructed using tree analysis, and validated with fivefold cross‐validation. A total of 574 patients with cancer were acutely (42%) or electively (58%) admitted 1733 times. The incidence rate of delirium was 3.5 per 100 admittances. Tree analysis revealed that the predisposing factors of an unscheduled admittance and a metabolic imbalance accurately predicted the development of delirium. In this group the incidence rate of delirium was 33 per 100 patients (1:3). The AUC of the model was 0.81, and 0.65 after fivefold cross‐validation. We identified that especially patients undergoing an unscheduled admittance with a metabolic imbalance do have a clinically relevant high risk to develop a delirium. Based on these factors, we propose to evaluate preventive treatment of these patients when admitted to the hospital in order to improve their quality of life.

## Introduction

Delirium is a common problem in hospital care, especially for patients with cancer as well as elderly and frail patients 1. It is a syndrome of brain dysfunction characterized by a disturbance in attention, awareness, and cognition, with a rapid onset that is caused by an underlying medical condition 2, 3. The occurrence of delirium depends on a combination of vulnerability (predisposing factors) and precipitating factors that trigger the development of delirium 4. Risk factors include aging, cognitive impairment, and a history of delirium, and screening for delirium in patients at risk may be of help to reduce suffering from delirium 5, 6, 7, 8, 9, 10, 11.

Data on the incidence and prevalence of delirium in literature range from a 5% prevalence rate upon admission to a geriatric hospital ward 12 up to an incidence rate of 88% in patients dying from cancer 8. This diversity in incidence and prevalence rates indicates that it is a serious problem for patients with cancer especially when terminally ill.

In recent publications it has been suggested that prophylactic treatment with antipsychotics should be considered to prevent delirium 13, 14, 15, 16, 17, but due to the varying incidence rates of delirium and the sometimes severe side‐effects of these medicines (primarily haloperidol) 18 there is reluctance to apply preventive treatment to all patients with cancer admitted to the hospital.

It would be of clinical significance if one could determine which patients are likely to develop delirium at admission in order to select patients who might benefit from prophylactic treatment.

In this study, the primary aim was to evaluate the occurrence of delirium and its risk factors in patients with cancer admitted to the hospital for treatment or palliative care in order to develop a prediction model to identify patients at high risk for delirium.

## Methods

This retrospective study was conducted in all patients with solid malignancies admitted to our medical oncology ward of the VUmc Cancer Center Amsterdam (CCA), VU University Medical Center, between Jan 1st 2011 and June 30th 2012. For each patient the following data were collected from medical charts: baseline characteristics, presence of delirium, and its risk factors throughout the entire admission. The study was conducted in accordance with the ethical standards of the responsible committee on human experimentation (institutional and national) and with the Helsinki Declaration of 1975, as revised in 2000. Because of the retrospective character of this study no additional informed consent could be obtained and a waiver was obtained from the medical ethical committee.

Screening for delirium was performed twice a week during three consecutive nursing shifts according to standard hospital procedures using the Delirium Observation Screening Scale (DOSS) 12. The DOSS is a validated 13‐item nurse‐rated screening instrument for delirium that is commonly used in Dutch hospitals. Scores range from 0 to 13 points, with ≥ 3 as a cut‐off for delirium. It has sensitivity and specificity rates of 92% and 82%, respectively 19. Delirium was recorded as present if the diagnosis was noted in the patient chart, or if the patient had a DOSS score ≥ 3 without a rejection of the delirium diagnosis written down in the chart.

The following risk factors were assessed: age > 70 years, alcohol or drug abuse, hearing, visual and/or cognitive impairment, history of delirium, high doses of opioids (>90 mg of oral morphine or equivalent), corticosteroids (>15 mg dexamethasone or equivalent), and/or sedatives (>2 mg lorazepam or equivalent), infections, postoperative state (until discharge of major surgery), constipation (note of constipation or note of delayed stool production for >48 hours), urinary retention, tumor burden and location, organ failure, and metabolic disturbances 4, 5, 6, 7, 8, 9, 10. In detail these risk factors are depicted in table 1. To avoid dependence the first admission with or without delirium for each patient was used for comparisons.

**Table 1:** Predisposing and precipitating factors for delirium

Predisposing factors
Age > 70 years
Alcohol or drug abuse
Hearing impairment
Visual impairment
History of delirium
Cognitive impairment

Precipitating factors		
Group	Factor	Cut‐off
High doses of psychotropic medication	Opioids	>90 mg morphine per day or equivalent
	Corticosteroids	>15 mg dexamethasone per day or equivalent
	Sedatives	>2 mg lorazepam per day or equivalent
Withdrawal	Alcohol	
	Sedatives	
	Other	
Infection	Fever	>38.5°C
	Sepsis	
	Urinary tract infection	
	Pulmonary tract infection	
	Other infections	
Postoperative state		
Constipation		
Urinary retention		
Intracranial neoplasm	Primary tumor	
	Metastasis	
	Carcinomatous meningitis	
Organ failure	Pulmonary failure	O_2_ sat < 88% or PO_2_ < 55 mm Hg
	Renal insufficiency	Creatinine > 312 mmol/L (3 x ULN) or creatinine > 3 × baseline
	Liver failure	Bilirubin > 60 mmol/L (3 × ULN) and/or ASAT > 150 U/L (5 × ULN) and/or ALAT > 200 U/L (5 × ULN)
	Cardiac failure	Requiring (prolonged) hospitalization
Metabolic disturbance	Calcium (corrected for albumin level)	<1.75 mmol/L > 3.1 mmol/L
	Sodium	<130 mmol/L >155 mmol/L
	Potassium	<3.0 mmol/L >6.0 mmol/L
	Glucose	<2.2 mmol/L >13.9 mmol/L
	Albumin	<20 g/L

### Development of a delirium prediction algorithm

Based on the predisposing factors and the grouped variables for the precipitating factors, a prediction algorithm was developed. For the development of this prediction algorithm, the groups were defined irrespective of the prevalence rates of the individual factors to limit the number of factors included (see Table 1). Grouped variables were defined positive if any of the factors in this group was present. Eastern Co‐operative Oncology Group (ECOG) performance status (0–2 vs. 3–4), palliative or curative treatment intention, and whether or not an admission was scheduled were also included in the prediction algorithm. For prediction models, it is recommended that the number of events should ideally be 10‐fold higher than the number of variables included in the model 20. Therefore, we enriched the database with 46 extra delirium cases that were consecutively diagnosed with DOSS screening between July 2012 and September 2013 (Table 2). These baseline characteristics of these cases did not significantly differ from the patients with delirium in the original dataset. The enriched database is suitable for the calculation of odds ratios and the identification of predictors, but not for the calculation of absolute risks 21. The absolute risks were calculated from the original database.

**Table 2:** Patients included in prediction algorithm

	Delirium *n *=	No delirium *n =*	Total *n =*
Study period	52	522	574
Extra delirium cases^a^	46	—	46
Total	98	522	620

a For adequate power in the development of the delirium prediction algorithm, data on the predisposing and precipitating factors of 46 patients who developed delirium between July 2012 and September 2013 were added to the original dataset. These data were only used for the development of this algorithm. Absolute risks at delirium reported in the article were calculated with the original dataset.

### Statistical analysis

Statistical evaluation of differences between nondelirious and delirious patients was performed with a *χ* ^2^‐test, the Fisher exact test, or the Student's *t*‐test, whenever appropriate. Because of the multiple comparisons an adjusted *P *= 0.01 was considered statistically significant. To create a delirium risk prediction algorithm that can be easily implemented in the clinic, we used a tree analysis method 22. All predisposing and the grouped precipitating factors for delirium were used in this tree analysis, irrespective of the *χ* ^2^‐test and students *t*‐test results, to predict the risk of developing delirium in subgroups of patients. The number of splits in the tree was chosen in order to minimize the cross‐validated prediction error. Fivefold cross‐validation was used for validation of the algorithm. For both the original and the cross‐validated model the area under the curve (AUC) was calculated. Data were collected in the web‐based database system OpenClinica version 3.1.2. Statistical tests were performed with SPSS version 20.0. The prediction algorithm was constructed with the software package R program Rpart (version 3.1).

## Results

A total of 574 individual patients were admitted 1733 times during the study period (mean 2.95 admittances per patient, ranging from 1 to 22 admissions per patient). Sixty delirium episodes were recorded for 52 individual patients, which resulted in a delirium incidence rate of 3.5 per 100 admittances. Nine percent of all patients admitted in this period developed delirium.

### Patient characteristics

Of all 1733 admittances, 1003 admittances (57.9%) were scheduled. The mean age of admitted patients was 60 years (SD 13.1) and 60% of the patients were male. Compared with patients who did not develop delirium, patients who developed delirium were significantly older (mean age of 59 vs. 67 years, respectively (*P *<* *0.001)), had a worse ECOG performance status at admittance, and more often received treatment with palliative intention or palliative care only. Ninety‐four percent of the patients who developed delirium had an unscheduled admittance, compared to 49% of the patients who did not develop delirium (*P *<* *0.001). In 10 of the 730 unscheduled admittances the indication for the admittance was suspected delirium (*n *=* *2), confusion (*n *=* *5), or drowsiness (*n *=* *3). Seven of these patients were diagnosed with delirium in the hospital. Patients with delirium stayed longer in the hospital, and the outcome was worse. In Table 3, these data are shown in detail.

**Table 3:** Patient characteristics

		Total *n *=* *1733	%	Total first admittance *n*=574	%	No delirium *n *=* *522	%	Delirium *n *=* *52	%	*P *= a	OR	95%CI of OR
Age	Mean (SD)	57.2 (14.2)		60.0 (13.1)		59.3 (13.3)		68 (8)		<0.001		
Gender	M:F	1076:657	62:38	345:229	60:40	313:209	60:40	32:20	62:39	0.83	1.068	0.595–1.919
Tumor type	Gastrointestinal	645	37	196	34	179	34	17	33			
	Genitourethral	369	21	129	22	119	23	10	19			
	Skin	50	3	34	6	25	5	9	27			
	Lung	2	0	2	0	2	0	0	0			
	Head & Neck	369	21	108	19	100	19	8	15			
	Brain	8	1	6	1	4	1	2	4			
	Sarcoma	120	7	20	4	20	4	0	0			
	Breast	97	6	53	9	48	9	5	10			
	Other	73	4	26	5	25	5	1	2	0.009		
ECOG performance status	0	223	13	96	17	96	18	0	0			
	1	964	56	262	46	252	48	10	19			
	2	385	22	146	25	127	24	19	37			
	3	132	8	56	10	40	8	16	31			
	4	29	2	14	2	7	1	7	14	<0.001		
Length of stay	Days (Median (IQR))	3 (2–6)		4 (2–7)		4 (2–7)		10 (5–16)		<0.001		
Outcome	Alive	1692	98	549	96	508	97	41	79			
	Deceased	41	2	25	4	14	3	11	21	<0.001	9.735	4.155–22.809
Treatment intention	Curative	626	36	174	30	166	32	8	15			
	Palliative	1107	64	400	70	356	68	44	85	0.01	2.067	1.079–3.958
Indication for admittance	Scheduled admittance	1003	58	270	47	267	51	3	6			
	*Chemotherapy*	885	51	209	36	208	40	1	2			
	Diagnostic procedures	68	4	40	7	39	7	1	2			
	Intervention	50	3	21	4	20	4	1	2			
	Unscheduled admittance	730	42	304	53	255	49	49	94	<0.001	17.102	5.264–550.560
	Clinical symptoms	535	31	251	44	209	40	42	81			
	Complication	195	11	53	9	46	9	7	14			
Delirium Type	Hyperactive							11	21			
	Hypoactive							20	39			
	Mixed							18	35			
	Unknown^b^							3	6			

a *P*–values of the comparison between the patients with delirium and the patients without delirium.

b Delirium type could not be determined from the information in the patient chart.

### Predisposing and precipitating factors

The most prevalent predisposing factors in this group of patients were age >70 and alcohol/drug abuse (21% and 8%, respectively). Although all factors were previously defined as predisposing factors, only age >70 significantly correlated with the development of delirium in univariate analysis (*P *<* *0.001).

The most prevalent precipitating factors were high doses of psychotropic medication, infection, constipation, and metabolic imbalance (25%, 22%, 19%, and 18%, respectively). The precipitating factors infection, constipation, urinary retention, organ failure, and metabolic imbalance were significantly related with the presence of delirium (*P *<* *0.001) (Table 4).

**Table 4:** Prevalence of predisposing and precipitating factors

		Total *n = *574	%	No delirium *n *=* *522	%	Delirium *n *=* *52	%	*P*=	Odds Ratio	95% confidence interval OR
Predisposing factors										
Age > 70	No	452	79	421	81	31	60			
	Yes	122	21	101	19	21	40	<0.001^a^	2.824	1.557–5.119
Alcohol/drug abuse	No	526	92	480	92	46	89			
	Yes	48	8	42	8	6	12	0.43	1.491	0.602–3.693
Hearing difficulty	No	556	97	508	97	48	92			
	Yes	18	3	14	3	4	8	0.07	3.024	0.958–9.549
Visual impairment	No	559	97	511	98	48	92			
	Yes	15	3	11	2	4	8	0.04	3.871	1.187–12.624
History of delirium	No	569	99	517	99	52	100			
	Yes	5	1	5	1	0	0	0.99	—	—
Cognitive impairment	No	562	98	514	99	48	92			
	Yes	12	2	8	2	4	8	0.02	5.354	1.555–18.431
Precipitating factors										
High doses of psychotropic medication	No	428	75	397	76	31	60			
	*Yes*	146	25	125	24	21	40	0.01	2.151	1.193–3.878
	Corticosteroids	43	7	43	8	0	0	0.03	—	—
	Sedatives	29	5	22	4	7	14	0.01	3.535	1.432–8.727
	Opioids	86	15	70	13	16	31	0.002^a^	2.870	1.513–5.445
Alcohol/drug withdrawal	No	561	98	511	98	50	96			
	*Yes*	13	2	11	2	2	4	0.33	1.858	0.401–8.619
	Alcohol	12	2	11	2	1	2	0.99	0.911	0.115–7.199
	Sedatives	0	0	0	0	0	0	—	—	—
	Other	2	0	1	0	1	2	0.17	10.216	0.630–165.771
Infection	No	448	78	425	81	23	44			
	*Yes*	126	22	97	19	29	56	<0.001^a^	5.524	3.062–9.966
	Fever	48	8	39	7	9	17	0.03	2.587	1.175–5.694
	Sepsis	16	3	9	2	7	14	<0.001^a^	8.849	3.148–24.879
	Urinary tract	30	5	22	4	8	15	0.003^a^	4.124	1.735–9.803
	Respiratory tract	26	5	17	3	9	17	<.001^a^	6.205	2.610–14.750
	Other	30	5	23	4	7	14	.01	3.368	1.370–8.279
Intracranial neoplasm	No	538	94	494	95	44	85			
	*Yes*	36	6	28	5	8	15	0.01	3.208	1.379–7.461
	Primary tumor	8	1	5	1	3	6	0.03	6.331	1.469–27.287
	Metastasis	24	4	19	4	5	10	0.06	2.816	1.006–7.885
	Carcinomatous meningitis	4	1	4	1	0	0	0.99	—	—
Post‐operative state	No	557	97	509	98	48	92			
	Yes	17	3	13	2	4	8	0.06	3.263	1.024–10.398
Constipation	No	468	82	433	83	35	67			
	Yes	106	19	89	17	17	33	0.006^a^	2.363	1.268–4.405
Urinary retention	No	560	98	514	99	46	89			
	Yes	14	2	8	2	6	12	0.001^a^	8.380	2.788–25.192
Organ failure	No	476	83	447	86	29	56			
	Yes	98	17	75	14	23	44	<0.001^a^	4.727	2.596–8.608
Liver failure	Not measured	77	13	70	13	7	13			
	No	437	76	401	77	36	69			
	*Yes*	60	10	51	10	9	17	0.09	1.971	0.898–4.326
	Bilirubin >60 mmol/L	19	4	15	3	4	21	0.08	2.849	0.903–8.983
	ASAT > 150	51	10	43	10	8	18	0.12	2.062	0.902–4.710
	ALAT > 200	24	5	21	5	3	7	0.47	1.469	0.421–5.131
Pulmonary insufficiency	Not measured	380	66	361	69	19	37			
	No	173	30	148	28	25	48			
	Yes	21	4	13	2	8	15	0.01	7.133	2.806–18.133
Renal failure	Not measured	15	3	15	3	0	0			
	No	543	95	497	95	46	88			
	Yes	16	3	10	2	6	12	0.002^a^	6.696	2.327–19.239
Cardiac failure	No	565	98	514	98	51	98			
	Yes	9	2	8	2	1	2	0.58	1.262	0.155–10.294
Metabolic imbalance	Not measured	20	3	20	4	0	0			
	No	453	79	428	82	25	48			
	*Yes*	101	18	74	14	27	52	<0.001^a^	6.538	3.599–11.878
	Calcium low	1/411	0	1/362	0	0/49	0	0.99	—	—
	Calcium high	8/411	2	4/362	1	4/49	8	0.009^a^	7.956	1.923–32.918
	Potassium low	30/550	5	21/498	4	9/52	9	0.001^a^	4.754	2.051–11.022
	Potassium high	5/550	1	1/498	0	4/52	8	<0. 001^a^	41.417	4.538–378.011
	Sodium low	25/544	5	18/493	4	6/51	12	0.02	3.519	1.330–9.311
	Sodium high	2/544	0	0/493	0	2/51	4	0.009^a^	—	—
	Hypoglycemia	1/395	0	0/350	0	1/45	2	0.11	—	—
	Hyperglycemia	24/395	6	19/350	5	4/45	9	0.32	1.700	0.551–5.240
	Albumin low	42/442	9	24/393	6	18/49	37	<0.001^a^	8.927	4.378–18.206

a *P* < 0.01.

### Delirium prediction algorithm

To determine the most relevant factors for the risk at delirium, a prediction algorithm by using tree analysis was developed using the enriched database. The optimum number of splits, with the lowest cross‐validated prediction error, was four.

**Figure 1:**
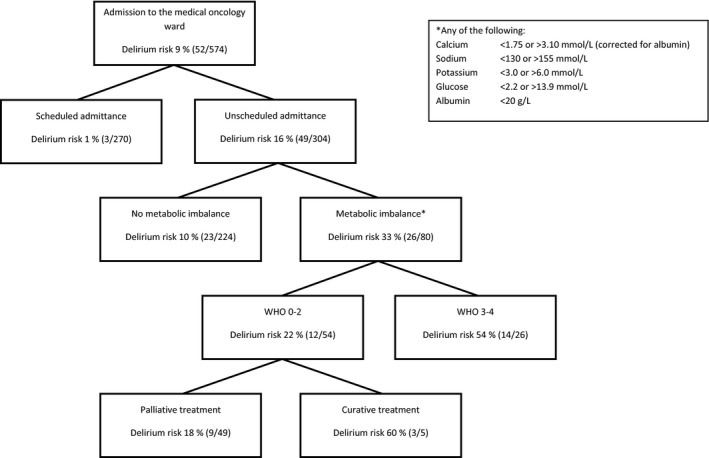
Delirium prediction algorithm. This risk is defined by the combination of factors mentioned in the boxes, starting with a baseline risk of 9% when a patient is admitted to the medical oncology ward. The * refers to the square in the corner of the figure in which the metabolic imbalances are defined.

**Figure 2:**
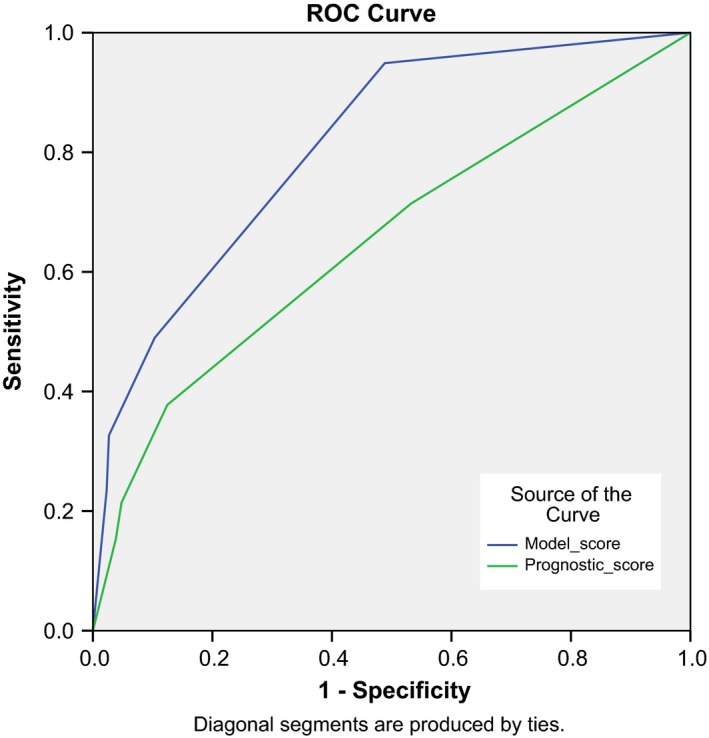
ROC curve of the prediction algorithm for delirium. These curves show the sensitivity and specificity of the different cut‐off points in the algorithm. AUC of the original model (blue line) is 0.81 and 0.65 for the cross‐validated model (green line).

The absolute risks in the decision tree, obtained by projecting the algorithm to the original, nonenriched dataset, are depicted in Figure 1. A patient admitted to the hospital ward has a risk of 9% to develop delirium (95% CI: 6.8–11.7%). The first factor that made a major distinction between a low risk at delirium (1.1% in the original dataset, 95% CI: 0.2–3.2%) and an intermediate risk at delirium (16% in the original dataset, 95% CI: 12–21%) was whether or not an admission was scheduled. Due to the very low risk at delirium (1:100), it was deemed unnecessary to make any further distinctions within the group with a scheduled admittance. In the group with an emergency admission, a further distinction could be made between patients who did or did not have metabolic imbalances. These patients had a delirium risk of 10% and 32.5%, respectively (95% CI: 6–15%, resp. 22–44%). In the group with an unscheduled admittance combined with a metabolic imbalance (delirium risk 1:3), ECOG performance status 0–2 versus ≥3, and curative versus palliative treatment intention were further splits. The AUC of this algorithm was 0.81 (Fig. 2 upper line). We evaluated predictive validity of the algorithm by fivefold cross‐validation. This provided a lower estimate for the AUC of 0.65 (Fig. 2 lower line), as the original algorithm estimates do not correct for uncertainty in the selection of predisposing and/or precipitating factors. The sum of the sensitivity and specificity was maximal at a cut‐off with a high specificity of 85%, and a lower sensitivity of approximately 40% in the cross‐validated algorithm. This cut‐off allows for identification of a subgroup of patients with a high risk at delirium. In the algorithm, the cut‐off is the distinction between patients with an unscheduled admittance with or without metabolic imbalances.

We evaluated whether the factors found in this algorithm were also predictive for different admissions of the same patient by comparing the prevalence of unscheduled admittances and metabolic imbalances within patients who had both an admittance with delirium and an admittance without delirium (27/52 patients with delirium). Admittances with delirium were significantly more often unscheduled than admittances without delirium (25/27 vs. 10/27, *P *<* *0.001). Metabolic imbalances were also more prevalent in the delirium admittance than in the admittance without delirium, but this difference was not statistically significant (12/27 vs. 6/27, *P *=* *0.08). During the admittance with delirium, patients had a higher chance to be in the high‐risk group according to the prediction algorithm (with the combination of an unscheduled admittance and metabolic imbalances), than during the admittance without delirium (12/27 resp. 4/27, *P *=* *0.02).

## Discussion

In this study, medical data from 574 patients during 1733 admittances were evaluated to determine the occurrence of delirium and its risk factors in patients admitted to the hospital for treatment or palliative care. We found a delirium incidence rate of 3.5 per 100 admittances and determined that 9% of all patients admitted in this period developed delirium. The most frequent predisposing factors in this group of patients were age >70 and alcohol/drug abuse, whereas the most frequent precipitating factors were high doses of psychotropic medication, infection, constipation, and metabolic imbalance.

Because of the large number of patients that were evaluated, it was possible to use both predisposing and precipitating factors to develop an algorithm that may be used in daily practice to identify patients with a high risk to develop a delirium.

The incidence rate of 3.5% per admittance in this evaluation is lower than the 16–18% reported on similar hospital wards 6, 9. A reason for the low incidence of delirium on this ward could be that half of the admittances were scheduled for patients to receive chemotherapy or undergo other interventions, as these patients have a low risk at delirium. In the study by Ljubisavljevic and Kelly (2003) these patients were not included, and it is likely that the proportion of scheduled admittances in the study by Gaudreau et al. (2005) was also smaller. When all scheduled admissions are excluded from our dataset, the incidence rate of delirium goes up to 7.8% (57/730), which is still lower than in the aforementioned studies. Another important reason that might explain this low incidence rate could be that the mean age of the patients admitted to this ward was 60 years (only 21% of the patients were aged ≥70 years) and even the elderly patients had a good cognitive performance status, as only 2.1% of all patients had a cognitive impairment.

The selected predisposing and precipitating factors were previously defined for their significant relationship with delirium, but this relationship was not confirmed for all of these factors in this study. This is most likely due to the low prevalence rates of these risk factors. In other studies logistic regression analysis to determine the influence of an individual factor on a patients risk at delirium had been used 8, 23. Although the results of these analyses indicate that a patient in whom a certain factor is present has a relatively higher risk at delirium, it does not provide the clinician with a clinical tool to clearly define the absolute risk that a specific patient has to develop delirium. Also, the effect of a combination of multiple predisposing and/or precipitating factors in the same patient is often not clear. Therefore, a prediction algorithm could be of significant clinical value to provide this information. Martinez et al. (2012) developed a prediction rule for patients admitted to the internal medicine ward 24. This prediction rule could not be applied to our medical oncology ward as the prevalence of some of the components of the prediction rule was too low (e.g., age > 85 years).

We developed an alternative algorithm in which patients with high risk for delirium are rapidly identified based on an emergency admittance combined with metabolic imbalances (delirium risk 1:3) (see Fig. 1). These factors are usually available upon admission of a patients with cancer and therefore this algorithm can be easily implemented in daily clinical practice. We here propose that based on this algorithm, patients could be selected for preventive treatment for delirium 12, 13, 14, 15, 16.

We do realize that our study has some limitations such as that it is a retrospective evaluation, the number of patients are rather limited to evaluate a high number (>10) of predisposing factors for delirium, and although it concerns only patients with cancer, tumor diagnosis is heterogeneous. On the other hand, the strength of this study is that no selection has been made for patients with cancer acutely admitted to the hospital and that the algorithm to determine the risk at a delirium can be easily implemented in daily practice.

In future studies, preventive treatment for delirium should be evaluated for its influence on the quality of life of patients, while taking in account the added risk of treatment‐induced toxicity of such a treatment strategy. In addition, as previously advocated by others, we also highly recommend screening of acutely admitted patients for delirium 25. The specificity for the cut‐off in our algorithm is high (85%), but the sensitivity is only 40%. This means that 60% of the delirium cases would be missed when only attention is being focused at patients in the high‐risk group. Therefore, while preventive treatment of patients identified by our algorithm with a high risk of delirium needs further evaluation, also screening for delirium symptoms in the other patients with an emergency admission should be considered.

In conclusion, delirium is a serious problem for patients with cancer admitted to the hospital. We identified that especially patients undergoing an unscheduled admittance with a metabolic imbalance do have a clinically relevant high risk to develop a delirium. Based on these factors, we propose to evaluate preventive treatment of these patients when admitted to the hospital in order to improve their quality of life.

## Conflict of Interest

The authors have no conflicts of interest to report.
